# Recommendations for athletes and COVID-19 vaccinations: A South African Sports Medicine Association (SASMA) position statement – Part 3

**DOI:** 10.17159/2078-516X/2021/v33i1a12557

**Published:** 2021-01-15

**Authors:** L Pillay, J Patricios, DC Janse van Rensburg, R Saggers, D Ramagole, P Viviers, C Thompson, S Hendricks

**Affiliations:** 1 Section Sports Medicine & Sport, Exercise Medicine and Lifestyle Institute (SEMLI), Faculty of Health Sciences, University of Pretoria, Pretoria, South Africa; 2 Wits Sport and Health (WiSH), Faculty of Health Sciences, University of the Witwatersrand, Johannesburg, South Africa; 3 Premier Soccer League COVID-19 Chief Medical Officer, PSL, South Africa; 4 Chief Medical Officer, Gauteng Lions Cricket, South Africa; 5 Medical Board Member, World Netball, Manchester, UK; 6 Campus Health Service, Stellenbosch University, South Africa; 7 Institute of Sport and Exercise Medicine, Division of Orthopaedic Surgery, Department of Surgical Sciences, Faculty of Medicine and Health Sciences, Stellenbosch University, South Africa; 8 FIFA Medical Centre of Excellence, South Africa; 9 Department of Paediatrics and Child Health, Charlotte Maxeke Johannesburg Academic Hospital, Johannesburg, South Africa; 10 Division of Physiological Sciences, Department of Human Biology, Faculty of Health Sciences, University of Cape Town, South Africa; 11 Health through Physical Activity, Lifestyle and Sport (HPALS) Research Centre, Department of Human Biology, Faculty of Health Sciences, University of Cape Town. South Africa; 12 Carnegie Applied Rugby Research (CARR) centre, Institute for Sport Physical Activity and Leisure, Leeds Beckett University, Leeds, England

**Keywords:** covid-19, vaccination, athletes, recommendations, sports

## Abstract

The COVID-19 pandemic initially led to the shutdown of all sport at a high cost to both the economy and athlete health. As risk-mitigating protocols evolved and were implemented, the playing of sport returned slowly to normal. The introduction of COVID-19 vaccinations enhances the means of protection and risk management for all. This South African Sports Medicine Association position statement provides recommendations for the vaccination of athletes.

Since the classification of COVID-19 as a pandemic by the World Health Organization (WHO) on March 11 2020,^[1]^ the entire world has been embroiled in dealing with this disease. Several countries around the world have experienced surges in infection numbers or “waves” of COVID-19 infections. These waves at separate times in different countries made planning travel and events difficult.^[2]^ It necessitated various levels of lockdown around the world, as determined by each country’s governmental responses to COVID-19 activity. All levels of sporting activities, from professional to school, have been affected for sustained periods. At the start of the pandemic, to reduce the transmission of COVID-19, lockdown meant that sports training and competition had to be cancelled.

The primary non-pharmaceutical interventions against the spread of COVID-19 infection remain: (1) hand hygiene, (2) respiratory hygiene, (3) mask-wearing, and (4) social and physical distancing (at gatherings in closed and open spaces). Many countries have allowed professional sport to return with strict infection-risk mitigating procedures in place, as approved by their respective governments. As our understanding of COVID-19 transmission advanced, and to reduce any further negative effects, professional sport was gradually re-introduced under highly controlled environments. These environments were termed ‘bio-secure environments’ (BSE) or bio-bubbles.

## Bio-secure environments (BSE)

Presently, certain sports in South Africa (such as cricket and rugby) are competing within bio-secure environments–commonly referred to as bio-bubbles. Bio-bubbles are possible in these sports as they have defined competitions (for example, the initial post-lockdown resumption of the Premier Soccer League (PSL) and the British and Irish Lions Rugby Tour)). Bio-bubbles cannot be achieved in a league setting in which teams play over a protracted period of time. Other negative aspects of bio-bubbles include the cost involved in hosting these competitions and the mental anxiety for all those involved.^[3],[4]^ As an example, football in South Africa completed the 2019/2020 season in a bio-bubble. The PSL completed the 2020/2021 season outside of a BSE but with strict risk-mitigating health protocols in place.

## Vaccinations

On 31 December 2020, the World Health Organisation (WHO) validated the first COVID-19 vaccination – an mRNA vaccine that was subsequently approved for emergency use.^[5]^ Subsequently, several vaccines are now approved for emergency use in South Africa. Since then, data have emerged validating the efficacy of vaccinations in decreasing both transmission and COVID-19 disease severity.^[6]^

The function of vaccines in the times of COVID-19 is to achieve the following:

1. Minimise the risk of contracting COVID-19.
2. If there is a breakthrough infection, it reduces the illness to mild symptoms and prevents progression to severe disease which may include hospitalisation and ventilatory support (such as High Flow Nasal Cannula Oxygen therapy and polymasks).
3. If there is a breakthrough infection, to reduce the length of time of being infective and reducing the spread of the virus.

COVID-19 vaccinations have been shown to significantly protect against poor disease outcomes.^[7]^ Apart from the primary aim of protecting athlete health, an important advantage of an athlete-dedicated vaccine programme is the facilitation of domestic and international travel, allowing major tournaments and leagues to recommence without the need for a bio-bubble. Being vaccinated may also reduce travel stress.^[8]^ Despite this however, challenges remain. All team members (athletes and support staff) should be fully vaccinated. If all team members are vaccinated, the risk of contracting and spreading COVID-19 in the team environment and community is significantly reduced. This limits the need for bio-bubbles. Certain countries may still require proof of a negative COVID-19 Polymerase Chain Reaction (PCR) test to avoid quarantining in that country.^[9]^

## Vaccination rollout in South Africa

The South African Vaccine Rollout Programme has been implemented in phases. Phase One began on 17 February 2021 and involved healthcare workers as part of the Sisonke Trial. ^[10],[11]^ The South African Health Products Regulatory Authority (SAHPRA) approved the Johnson and Johnson COVID-19 vaccine on 31 March 2021.^[12]^ Further vaccines have subsequently been approved by SAHPRA (Pfizer, Sinovac) –see Table 1. Some athletes competing in major international events, such as the Olympic Games and the British and Irish Lions Rugby Tour, were also enrolled in this trial. The targeted rollout approach for the general population was then extended to the sixty years and older age groups first, to limit severe disease and death. Thereafter, individuals with chronic comorbid medical conditions (e.g. diabetes and hypertension) received the vaccination. Phase Two involved essential workers (teachers, police members), and those in congregated settings (for example, religious leaders). The age limit was later expanded to those over 40 years of age. Phase Three involved targeting the remainder of the population, in order to achieve a national target of 67%. Most athletes fell into this latter age group.

The nature of professional sport involves training and competing at high-intensity levels. Team sports involve not only interaction with team mates, but also the technical, medical and administrative staff. Individual sports involve athlete interaction with coaching and medical staff. Complying with non-pharmaceutical interventions (proper mask-wearing, hand sanitising and respiratory hygiene) as risk mitigating measures remain vital. Vaccination of athletes further enhances protection against contracting the COVID-19 virus and should be strongly encouraged.

Table 1 displays the availability and approval status of vaccinations in South Africa. It is important to note that presently SAHPRA has approved the Pfizer and Johnson and Johnson vaccines which are part of the nationwide rollout. These have been approved for those eighteen years and older. Sinovac has been approved for children twelve years and older. Others have been approved, but not rolled out in South Africa (Astra Zeneca) and others are in the process of being approved.

## Recommendations for the vaccination of athletes

1. Allow flexible training plans for three days post-vaccination, as one cannot predict when vaccine-related side effects will occur. Common side effects include headaches, myalgia, fatigue, fever and pain at the site of injection which can typically last two to three days. ^[13],[14]^
2. On the day of vaccination, Paracetamol (given at one gram three times a day) can be taken to reduce side effects, if these are experienced.
3. If there is a concern regarding allergies and an athlete is prone to severe allergic reactions, it is advisable to visit a vaccination site where medical staff are available to attend to these allergic reactions.^[15]^
4. Exercise may be performed on the day of vaccination but should be limited to low-intensity sessions. Avoid medium- to high-intensity exercise for at least three days.^[13]^
5. Should an athlete experience any vaccine-related side effects, then exercise should be avoided until these symptoms resolve.
6. Should an athlete experience persistent or worsening vaccine-related side effects continuing for five days post-vaccination, then an active COVID-19 infection must be excluded with a PCR test. This should be reported to SAHPRA. The athlete should consult a medical doctor.^[16]^
7. Should an athlete receive the Pfizer vaccine, and within 7–14 days post-vaccination experience fever, lethargy, shortness of breath and chest pain, myocarditis/pericarditis these symptoms must be considered and the athlete excluded from physical activities (usually with haematological investigations and an electrocardiogram – ECG). It may also warrant a referral to a cardiologist.^[17]^
8. If an athlete has recently been infected with COVID-19, he/she should be vaccinated at least four weeks after de-isolation or symptom resolution.^[18]^
9. Presently, vaccine choice is limited due to SAHPRA approval and availability. In principle, all vaccines can be used; indeed, any vaccination is better than none. Each vaccine’s efficacy and pitfalls should be considered if options are available.
10. As the science evolves, booster vaccinations may become available. This will be determined by the time of the vaccine effect waning and the type of vaccine initially received. There will ongoing and evolving evidence and approvals as more research is undertaken.

## Considerations for special populations

Youth: Sinovac has been recently approved by SAHPRA for those 12 years and older.^[19]^

Pregnancy: Vaccinations can be administered to pregnant (at any stage of pregnancy) and lactating mothers.^[20]^

## Conclusion

Vaccinating the athletic population is vital for the continuation of team and individual sport in a safe manner. Vaccinations reduce the risk of contracting and transmitting COVID-19, and allows for events to continue uninterrupted.

## Figures and Tables

**Table 1:** Available vaccines and status of approval in South Africa by South African Health Products Regulatory Authority (SAHPRA)^[13]^

Manufacturer	Vaccine	Platform	Used in South Africa
**Vaccines approved by SAHPRA for use in South Africa**			
AstraZeneca	ChAdOx1 nCov-19 (AZD1222/Covishield)	Viral vector	No
Pfizer/BioNTech	BNT-162b2	mRNA	Yes
Johnson & Johnson	AD26.COV2.S	Viral vector	Yes
Sinovac	Coronavac	Inactivated	Not as yet

**Applications submitted and awaiting approval from SAHPRA**			
Gamaleya Research Institute	Sputnik V	*rolling review by SAPHRA	No

**No application for approval in South Africa (but have WHO Emergency Use Listing)**			
Sinopharm	BBIBP-CorV	Inactivated	N/A
Moderna	mRNA-1273	mRNA	N/A
Novavax	NVX-CoV2373	Recombinant protein	N/A
